# How Stress Mindset Mitigates Burnout: The Role of Hope in Work–Family Conflict Among Chinese Teachers

**DOI:** 10.3390/bs16020186

**Published:** 2026-01-27

**Authors:** Qianfeng Li, Bohan Li, Caner Zhao, Shaobei Xiao

**Affiliations:** 1 School of Psychology, Hainan Normal University, Haikou 571158, China; bohanli0124@gmail.com (B.L.); zhaoqi1329773200@gmail.com (C.Z.); xiaoshb@hainnu.edu.cn (S.X.); 2 Adolescent Psychological Development and Education Center of Hainan, Hainan Normal University, Haikou 571158, China

**Keywords:** stress mindset, burnout, work–family conflict, hope

## Abstract

Teacher burnout is a pressing global issue with significant implications for educational quality. Although work–family conflict (WFC) is a well-documented cause of teacher burnout, the psychological mechanisms underlying this relationship remain insufficiently understood and warrant examination through the lens of individual resources and positive psychology. This study investigated the relationship between work–family conflict (WFC) and burnout among Chinese elementary and middle school teachers, with a specific focus on the mediating and moderating roles of hope and a stress-is-enhancing mindset. Data were collected from 452 teachers (including 355 females) using well-validated scales. The results revealed that: (1) WFC was found to be directly and positively associated with burnout, as well as indirectly associated through the mediating role of hope. (2) A stress-is-enhancing mindset moderates the negative association between WFC and hope. Specifically, the negative association between WFC and hope was significantly weaker among teachers with a high level of this mindset compared to those with a low level. These findings suggest that fostering hope and cultivating a stress-is-enhancing mindset can mitigate burnout risk, pointing to a viable pathway for promoting occupational well-being by developing teachers’ psychological resources.

## 1. Introduction

Burnout refers to a long-term reaction to stressors within the workplace, including emotional and interpersonal stress. It includes three key aspects: emotional exhaustion, depersonalization, and diminished professional efficacy (Maslach & Leiter, 2016). The issue of teacher burnout is worthy of global attention, as it can lead to multiple negative outcomes. Numerous studies have demonstrated that burnout may lead to a brain drain in the education industry (Rajendran et al., 2020), damage teachers’ physical and mental health (Chang, 2009; Olivier & Venter, 2003), reduce their job satisfaction and motivation (Skaalvik & Skaalvik, 2020), and even threaten students’ academic achievement and adjustment (Herman et al., 2018). The severity of professional burnout among Chinese teachers is starkly reflected in international comparisons, where they ranked second for emotional exhaustion among educators from 35 countries (García-Arroyo et al., 2019). Chinese teachers, especially those in elementary and secondary schools, operate in a challenging environment characterized by educational reforms such as the “Double Reduction” policy, and exceptionally high expectations from society and parents. This situation generates significant occupational stress that severely impacts their family lives, thereby increasing their vulnerability to long-term professional burnout (Zhong et al., 2025). Empirical research found work–family conflict (WFC) to be the principal direct contributor to burnout. (Zábrodská et al., 2018).

Moreover, in the Chinese cultural context, familism and normative role expectations place considerable emphasis on fulfilling both family and professional obligations. Such culturally specific pressures may intensify the impact of work–family conflict on psychological resources (Y. Li et al., 2024), making future-oriented personal resources like hope particularly salient for sustaining well-being. However, it remains unclear how WFC relates to burnout within the scope of primary and secondary school teachers.

### 1.1. Work–Family Conflict (WFC) and Burnout

Work–family conflict, also known as work–family interference, occurs due to the conflict of role expectations of work and family because of their different demands. According to conflict theory, the competing demands of work and family often result in tension and role strain because of their different expectations and norms (Greenhaus & Beutell, 1985; Byron, 2005). This incompatibility creates a structural foundation for WFC. Therefore, WFC involves two key aspects: work interference with family (WIF) and family interference with work (FIW). WIF refers to the extent to which experiences and demands at work negatively affect family life. FIW refers to the impact of family obligations on work performance (Frone et al., 1992). Work–family conflict among teachers contributes to role strain and ambiguity, which may have negative impacts on balancing professional and personal responsibilities effectively, ultimately resulting in lower job satisfaction, reduced organizational commitment, and diminished overall well-being (Nart & Batur, 2014; X. Li et al., 2021).

According to the Conservation of Resources (COR) theory, work–family (WFC) is a key reason for burnout because of resource depletion mechanisms. When resource demands exceed capacity, stress and burnout ensue. COR theory indicates that individuals are motivated to obtain, retain, and protect valued resources such as time, energy, and emotional well-being. Based on the core principle of Conservation of Resources (COR) theory, individuals’ experience of resource change is fundamentally asymmetric: resource loss provokes greater attention and reaction than does an equivalent resource gain (Hobfoll et al., 2018). To prevent further loss, individuals tend to invest additional resources; if resources are not replenished over time, they may enter a “resource loss spiral,” eventually leading to exhaustion and psychological distress. WFC accelerates resource loss by forcing individuals to distribute these limited assets across conflicting domains, leading to heightened stress and reduced capacity for recovery (Hobfoll, 1989; Grandey & Cropanzano, 1999; Halbesleben et al., 2014). In the teaching context, high workloads, administrative pressures, and familial responsibilities exacerbate resource strain (Hobfoll et al., 2018). This often results in emotional exhaustion, an essential component of burnout. Moreover, emotional exhaustion may not only result from WFC but also intensify it, forming a self-reinforcing cycle of stress and fatigue (Boles et al., 1997; Zhu et al., 2021). When the emotional demands of both roles surpass available resources, burnout becomes increasingly likely (Brotheridge & Lee, 2002; Liu & Cheung, 2015).

**Hypothesis** **1.** *Work–family conflict is positively associated with burnout.*

### 1.2. The Mediating Role of Hope

As a core dimension of psychological capital, hope, defined as pathway thinking to identify solutions and the agency thinking to pursue them (Snyder, 2002), functions as a critical personal resource, particularly in professions characterized by persistent role strain and long-term commitment, such as teaching. Prior research has shown that hope is negatively associated with burnout (Pharris et al., 2022). Consistent with this protective role and grounded in COR theory, empirical research suggests that hope helps replenish psychological resources depleted by work–family conflict, thereby buffering individuals against job burnout (Pu et al., 2017). COR theory posits that persistent resource depletion, without adequate recovery or gain, precipitates a “resource loss spiral” that substantially increases burnout risk. Within this framework, the depletion of hope may serve as an important mediating mechanism through which work–family conflict initiates a resource loss spiral leading to burnout. Accordingly, the present study focuses specifically on hope to provide a more precise examination of this mediating mechanism. Although prior studies have emphasized the mediating role of overall psychological capital (e.g., Y. Wang et al., 2012, in a sample of doctors), the present study focuses on hope to clarify its specific role within this process.

Building on this theoretical foundation, the present study further argues that hope is particularly relevant for understanding teacher burnout arising from work–family conflict because it directly targets future-oriented goal pursuit under persistent role strain. Specifically, the pathways component enables individuals to generate alternative strategies when obstacles arise, enhancing adaptability and helping maintain alignment between job demands and personal aspirations, whereas the agency component sustains motivation and goal-directed confidence, allowing individuals to persist in their efforts despite ongoing difficulties. As a result, employees with higher levels of hope are better equipped to cope with work–family challenges, apply effective problem-solving strategies, and mitigate resource depletion, ultimately experiencing lower levels of burnout (Djudiyah et al., 2023; Yavas et al., 2013). Based on research, this study proposes the following hypothesis:

**Hypothesis** **2.** *Hope mediates the relationship between work–family conflict and burnout. Specifically, higher work–family conflict is associated with lower hope, which in turn is associated with higher burnout.*

While identifying the mediating role of hope clarifies the process through which work–family conflict impacts burnout, it is equally important to understand the conditions under which this process may be amplified or attenuated. Therefore, we introduce a stress-is-enhancing mindset as a critical personal resource and boundary condition that potentially moderates these relationships.

### 1.3. The Moderating Role of Stress-Is-Enhancing Mindset

Stress-is-enhancing mindset can reduce the adverse results of work–family conflict (Hammond et al., 2021). Following the stress mindset theory (Crum et al., 2013), individuals hold general beliefs about the nature of stress, ranging from a ‘stress-is-debilitating’ mindset to a ‘stress-is-enhancing’ mindset. In the present study, we focus specifically on the ‘stress-is-enhancing’ mindset, the belief that the experience of stress has positive consequences for performance, learning, and growth. Throughout this study, the term ‘stress-is-enhancing mindset’ refers to this specific positive pole of the broader stress mindset continuum, which is particularly relevant in coping with work–family challenges. Research has demonstrated that a stress-is-enhancing mindset contributes to more positive outcomes by influencing individuals’ behavioral, psychological, and physiological responses (Hammond et al., 2021). Specifically, A stress-is-enhancing mindset can increase individuals’ positive emotions and enhance cognitive flexibility, especially when facing stress (Crum et al., 2017). Additionally, individuals with this mindset tend to experience relatively low stress levels and are more motivated in the workplace. They also exhibit more proactive coping strategies when anticipating high work demands and demonstrate better job performance (Casper et al., 2017).

Within the Conservation of Resources framework, a stress-is-enhancing mindset is conceptualized as a cognitive personal resource that shapes how individuals appraise stress, orienting attention toward potential resource gains rather than losses. Research shows that a stress-is-enhancing mindset moderates the relationship between stressors and various outcome variables (Hammond et al., 2021). For example, a study involving nurses found that a stress-is-enhancing mindset weakened the relationship between job demands and burnout (Zhou et al., 2024). According to Hahm (2016), stress-is-enhancing mindset in the work context moderates the association between job stress and key components of burnout, specifically exhaustion and cynicism. Moreover, research shows that a stress-is-enhancing mindset moderates the relationships between work–family conflict and important outcomes, including job satisfaction and turnover intentions (Hammond et al., 2021). Furthermore, the stress-is-enhancing mindset moderates adolescents’ negative feelings caused by life events. Individuals who perceive stress as a negative factor tend to experience more significant psychological distress when facing high levels of adversity (Park et al., 2018). As evidence shows that stress can impact individuals’ responses to stress (Crum et al., 2017), it is likely to be a moderator in the association between work–family conflict and burnout.

**Figure 1:**
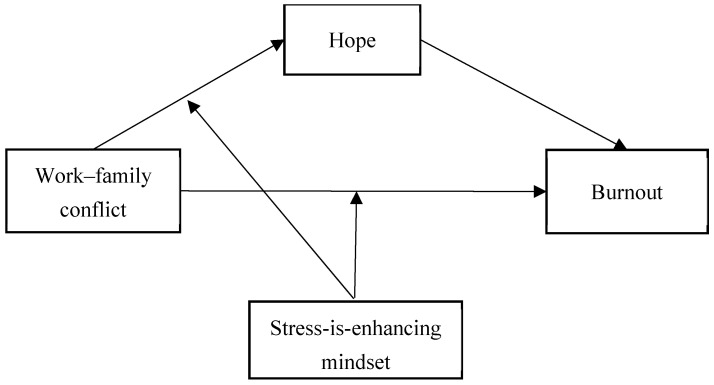
Proposed moderated mediation model.

The present study conceptualizes hope and stress-is-enhancing mindset as functionally distinct but theoretically complementary personal resources within COR theory. Specifically, hope captures the internal motivational process of resource loss by reflecting the erosion of future-oriented agency and pathways thinking under work–family conflict, thereby mediating its effect on burnout. In contrast, a stress-is-enhancing mindset serves as a higher-order cognitive moderating resource that explains when and for whom this resource loss process is attenuated. This study aims to examine a moderated mediation model, investigating whether a stress-is-enhancing mindset buffers both the direct and indirect (via hope) associations between work–family conflict and burnout. The conceptual framework guiding our investigation is demonstrated in Figure 1.

**Hypothesis** **3a.** *A stress-is-enhancing mindset moderates the direct association between work–family conflict and burnout, such that the positive relationship between work–family conflict and burnout is weaker among individuals with a stronger stress-is-enhancing mindset.*

**Hypothesis** **3b.** *A stress-is-enhancing mindset moderates the indirect relationship between work–family conflict and burnout through hope, such that the indirect effect of work–family conflict on burnout via reduced hope is attenuated among individuals with a stronger stress-is-enhancing mindset.*

## 2. Materials and Methods

### 2.1. Participants

The sample consisted of 452 teachers from seven primary and secondary schools located in Beijing and Chongqing, China. Among the participants, 196 (43.4%) were from schools in rural areas (villages or townships where agriculture is the primary economic activity), and 256 (56.6%) were from schools in urban areas (urban districts, counties, or towns dominated by non-agricultural activities). The age of participants spanned from 22 to 58 years (Mage = 39.4, SD = 8.62), including 355 females (78.5%) and 97 males (21.5%).

### 2.2. Procedure

A multistage sampling strategy was adopted to recruit participants from primary and secondary schools. Initially, regions were identified using a convenience sampling technique. Beijing, Henan, Jiangsu, and Chongqing were chosen to represent China’s central, eastern, and western regions based on the country’s economic zone division. Subsequently, one county from each city was chosen for further investigation. Finally, within each selected county, contact persons from different schools were identified for sampling. In schools with large populations of teachers (over 60), we randomly selected 50–70% of them as participants. In smaller schools with a moderate number of teachers (30–60), a group sampling was conducted to ensure sufficient representation. For small and some rural schools, convenience sampling was applied to recruit participants. Before answering electronic questionnaires, all participants signed the informed consent, which took approximately 15 min. Upon completion, they received 20 yuan as an expression of gratitude. As the work–family conflict measure was administered exclusively to participants in Beijing and Chongqing, the analyses related to this variable are based solely on data from these two municipalities.

### 2.3. Measures

#### 2.3.1. Demographic Characteristics

Data on each participant’s age, sex, years of teaching, educational level, marital status, monthly income, and health status were collected.

#### 2.3.2. Work–Family Conflict

Teachers’ experiences of work–family conflict were measured using a validated work–family conflict scale. The scale was adapted from Gutek et al. (1991) and further modified by Gao and Zhao (2014), which included two subscales of work-to-family interference and family-to-work interference. The scale consists of ten items, with five items addressing work-to-family interference (e.g., “Work takes time away from spending time with my family”) and five items addressing family-to-work interference (e.g., “I often have to think about family matters while at work”). Participants rated each item on a five-point Likert scale, with one representing “strongly disagree” and five representing “strongly agree.” The average score was calculated, with higher scores indicating greater work–family conflict. The Chinese version has been adopted in prior studies with Chinese teachers and has demonstrated acceptable reliability and construct validity (e.g., Wei et al., 2024). In the present study, the Cronbach’s α of this scale was 0.83.

#### 2.3.3. Hope

An adapted Chinese version of the Psychological Capital Scale (Luthans et al., 2005) was utilized to evaluate teachers’ psychological capital in relation to their career development. The scale consists of four dimensions: self-efficacy, hope, resilience, and optimism. Six items are concluded in each dimension, for a total of 24 items. To assess the level of hope, the data from the hope subscale were included in the analysis of the current study. Participants rated each item on a 6-point Likert scale (1 = strongly disagree, 6 = strongly agree). The average scale score was calculated, with higher scores indicating higher levels of psychological capital. The scale has been widely used for measuring psychological capital across various occupational groups in China (e.g., Wei et al., 2024). In the present study, the Cronbach’s α of the hope dimension was 0.94.

#### 2.3.4. Stress-Is-Enhancing Mindset

This study used the Stress Mindset Measure to assess how much teachers believe stress is beneficial or harmful (Crum et al., 2013). The scale contains eight statements. The example of items is “The effects of stress are negative and should be avoided.” Participants rated their extent of agreement on each statement on a 5-point Likert scale (“0” = Strongly Disagree, “4” = Strongly Agree). The items, response format, and scoring procedure fully correspond to the original instrument. Mean scores were calculated, with higher scores showing a stronger stress-is-enhancing mindset. The Chinese version has been adopted in prior studies with Chinese samples and has demonstrated acceptable reliability and construct validity (e.g., Wu et al., 2022). In the present study, the internal consistency (Cronbach’s α is 0.72) falls within the acceptable range for psychological constructs in applied field research. Additionally, we performed item-level analyses. Item–total correlations indicated that all items contributed meaningfully to the overall scale (r value ranged from 0.5 to 0.6), with no item showing unusually low correlations that would suggest the need for item removal.

#### 2.3.5. Burnout

Teacher burnout was assessed using the Educator Burnout Inventory (EBI) developed by G. Wang et al. (2003), which comprises 21 items across three dimensions: exhaustion (e.g., feeling emotionally depleted at the end of the workday), depersonalization (e.g., interacting with students in an impersonal or detached manner), and diminished personal accomplishment. Participants rated the frequency of their experiences on a seven-point scale, ranging from 1 (a few times per year) to 7 (every day). Mean scores were calculated, with higher values indicating greater levels of burnout. The Chinese version has been adopted in prior studies with Chinese teachers and has demonstrated acceptable reliability and construct validity. In the present study, the Cronbach’s α is 0.90.

### 2.4. Data Analysis

First, this study used Harman’s single-factor test to evaluate the extent of common method bias. Descriptive statistics for demographic variables and bivariate correlations among key constructs (work–family conflict, burnout, hope, stress-is-enhancing mindset) and demographic factors were subsequently calculated using SPSS 27.0. Subsequently, the mediation model was tested using regression and a bootstrapping approach with the assistance of the PROCESS macro. An indirect effect was deemed statistically significant if the 95% bootstrapped confidence interval excluded zero. To investigate moderation, Model 8 of the PROCESS macro was applied to test whether the stress-is-enhancing mindset moderated the mediation pathway. Additionally, this study used simple slope analysis to better illustrate the interaction effect.

## 3. Results

### 3.1. Descriptive Statistics

Participants’ demographic characteristics and the means and standard deviations of the studied variables are shown in Table 1. The majority of participants were married (79.4%), with an undergraduate (78.3%) or postgraduate (17.3%) degree as their highest educational level. The mean years of teaching were 17.11 (SD = 10.06), and the mean monthly income was 9234.85 (SD = 1807.16). This study used Harman’s single-factor test to assess common method bias. Results showed that the first unrotated factor was in proportion to 21.3%, indicating that common method bias was not a significant concern in this study. Additionally, the normality of all major study variables was assessed by examining their skewness and kurtosis. The skewness values ranged from 0.03 to 0.90, and the kurtosis values ranged from 1.0 to 3.3, both of which fall well within the conservative thresholds for regression models (Matore & Khairani, 2020). This confirms that the assumptions for parametric analysis were met.

### 3.2. Correlations

Correlations among these studied variables were demonstrated in Table 2. Burnout was positively associated with work–family conflict (r = 0.49, *p* < 0.001), suggesting that work–family conflict exacerbated burnout. Burnout was inversely related to the stress-is-enhancing mindset (r = −0.36, *p* < 0.001), hope (r = −0.44, *p* < 0.001), and health status (r = −0.22, *p* < 0.001), indicating that higher levels of burnout were associated with lower levels of hope, a weaker stress-is-enhancing mindset, and poorer perceived health. Work–family conflict exhibited an inverse relationship with hope (r = −0.15, *p* < 0.05), stress-is-enhancing mindset (r = −0.24, *p* < 0.001), and health status (r = −0.32, *p* < 0.001), indicating that individuals going through more work–family conflict had less hope and tended to view stress as harmful. Hope demonstrated a positive relationship with the stress-is-enhancing mindset (r = 0.31, *p* < 0.001), monthly income (r = 0.16, *p* < 0.05), and health status (r = 0.14, *p* < 0.05), suggesting that hopeful individuals were more likely to keep a positive view of stress and report reduced experiences of work–family conflict. The gender and the educational level were not significantly associated with any of the study variables.

### 3.3. Mediation Analysis of Hope

The current study explored the effect of hope on the pathway from work–family conflict to burnout. Results were demonstrated in Table 3, which indicated that work–family conflict was significantly and positively related to burnout both directly (B = 0.657, *p* < 0.001, 95% CI [0.537, 0.776]) and indirectly via hope (B = 0.060, SE = 0.033, 95% CI [0.001, 0.134]), after controlling for years of teaching, married status, monthly income, and health status. These findings suggested that higher levels of work–family conflict were associated with lower levels of hope, which in turn were linked to greater burnout. Gender was examined as a demographic variable but was not included as a covariate in the main analyses due to its nonsignificant associations with the focal variables.

### 3.4. Moderation Analysis of Stress-Is-Enhancing Mindset

The moderated mediation model was tested using the PROCESS macro. Findings were demonstrated in Table 4, which revealed that the stress-is-enhancing mindset did not significantly moderate the direct relationship between work–family conflict and teacher burnout (B = 0.152, 95% CI [−0.012, 0.315]). However, it moderated the relationship between work–family conflict and hope (B = 0.171, 95% CI [0.011, 0.330]), thereby influencing the indirect path through hope. Specifically, work–family conflict led to a significant loss of hope when the stress-is-enhancing mindset was at a low level (B = −0.170, SE = 0.075, 95%CI [−0.317, −0.023]), but not when it was at a high level (B = 0.010, SE = 0.072, 95%CI [−0.132, 0.152]).

**Figure 2:**
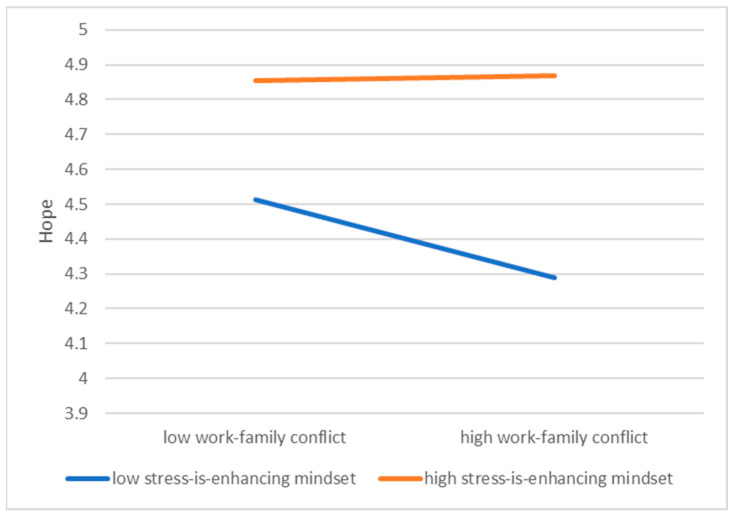
The interaction between work–family conflict and stress-is-enhancing mindset on teachers’ hope.

**Figure 3:**
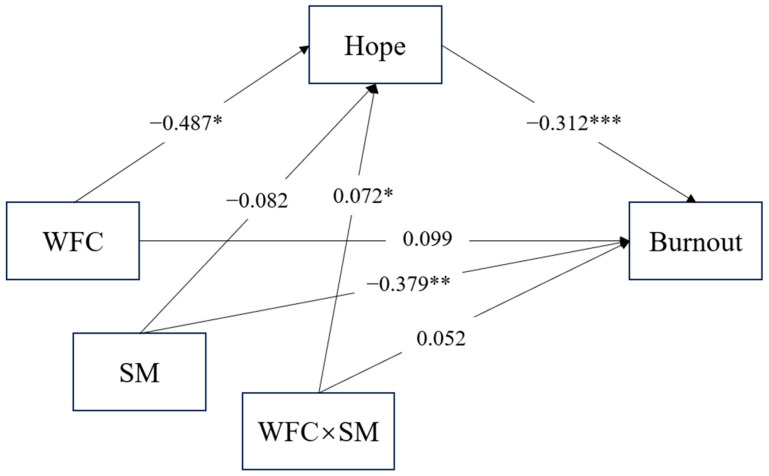
Coefficients of pathways in the final model. WFC = work–family conflict; SM = stress-is-enhancing mindset. Note: * *p* < 0.05, ** *p* < 0.01, *** *p* < 0.001.

To further explore the interaction of these variables, a simple slope analysis was conducted. As illustrated in Figure 2, the relationship between hope and work–family conflict changed as individuals maintained a high or low level of stress-is-enhancing mindset. Among those with a high level of stress-is-enhancing mindset, the work–family conflict could not damage their hope, resulting in an attenuated indirect effect of this pathway. In contrast, the direct effect of work–family conflict on burnout remained robust and statistically significant across all levels of stress-is-enhancing mindset (*p* < 0.001). The coefficients of pathways in the final model were depicted in Figure 3.

## 4. Discussion

The present findings indicate that work–family conflict is positively associated with burnout, with hope functioning as a mediator in this relationship. Moreover, a stress-is-enhancing mindset serves as an effective moderator by buffering the detrimental effect of work–family conflict on hope. Beyond the existing literature on psychological capital and burnout, this study contributes by integrating a stress-is-enhancing mindset into the work–family conflict–burnout process, thereby specifying both the process (resource depletion via hope) and the boundary condition (stress-is-enhancing mindset) through which work–family conflict translates into burnout in Chinese educational contexts. Practically, the results suggest that supporting teachers under high work–family demands may benefit from approaches that enhance goal clarity and perceived pathways (e.g., mentoring or career planning) and encourage more adaptive interpretations of stress. Although evidence for such interventions among teachers remains emergent, these findings point to promising directions for future development rather than immediately prescriptive solutions.

Consistent with previous research (Pu et al., 2017), the finding suggests that burnout is significantly linked to work–family conflict, suggesting that teachers with higher work–family conflict are likely to experience greater levels of burnout. Moreover, the finding indicates the underlying mechanisms driving this effect, specifically highlighting hope as a mediator and a stress-is-enhancing mindset as a moderator. These findings provide further support for existing literature (Gustafsson et al., 2010; Gustafsson et al., 2013; Szcześniak et al., 2024; Herbert, 2011). This result is consistent with the COR Theory, which asserts that work–family conflict depletes teachers’ psychological resources, making them more susceptible to burnout. These findings suggest that the impact of job stress on burnout may operate through the depletion of hope as a crucial psychological resource (affecting emotional, cognitive, and motivational dimensions of burnout) (Gustafsson et al., 2013). In this way, reduced hope may be linked to heightened job stress (e.g., work family conflict), which may further relate to higher levels of burnout. In addition, although the indirect effect of work–family conflict on burnout via hope was statistically significant, the magnitude of this effect was small. This may imply that hope may function as a modest psychological resource that can help buffer the impact of work–family conflict on burnout. Practically, future interventions aimed at fostering hope may offer incremental benefits when integrated into broader support systems, but they are unlikely to fully offset the effects of substantial work–family strain. Importantly, these findings should not be interpreted as implying that individual-level psychological resources can substitute for organizational or structural efforts to reduce work–family conflict.

Furthermore, our findings indicate that a stress-is-enhancing mindset buffers the negative impact of work–family conflict on hope. In other words, stress-is-enhancing mindset significantly moderates the association between work–family conflict and hope, which in turn conditions the indirect relationship between work–family conflict and burnout through hope. Specifically, individuals with higher levels of stress-is-enhancing mindset experience a weaker negative effect of work–family conflict on hope, thereby reducing the indirect impact of work–family conflict on burnout. These results clarify the mechanism through which stress-is-enhancing mindset can help mitigate burnout, consistent with prior research (Hahm, 2016; Zhou et al., 2024). These findings suggest a process-specific moderating effect rather than a general buffering of burnout. That is, stress-is-enhancing mindset does not directly alter the strength of the work–family conflict–burnout link, but instead shapes how work–family conflict translates into resource depletion in the form of reduced hope, which subsequently relates to burnout.

To better understand this result, we considered two explanatory perspectives. First, a stress-is-enhancing mindset enables individuals to maintain positive emotions and proactively adopt coping strategies. When individuals view stress in a gain-oriented way, believing it can have beneficial effects, they experience less stress and their emotional responses become more positive (Chen & Hou, 2021). Positive emotions help alleviate the emotional burden caused by work–family conflict, thereby reducing the erosion of hope. Additionally, stress can be defined as an opportunity rather than a threat to individuals with a stress-is-enhancing mindset. They may view work–family conflict as a challenge, prompting them to adopt more proactive coping strategies and focus on achieving goals and completing tasks (Mansell & Turner, 2023). This may allow them to address problems at the source, creating a positive feedback loop. Second, the COR theory suggests that individuals facing a higher threat of resource loss than gain are more vulnerable to work–family conflict. (Oren & Levin, 2017). Both a stress-is-enhancing mindset and hope are valuable personal resources. When facing stress from work–family conflict, these resources can assist individuals in managing the conflict. By not feeling resource depletion when confronted with conflict, individuals reduce the likelihood of experiencing work burnout.

There are several limitations in the current study. First, among various personal resources, this study chose to focus solely on hope. Future research examining additional personal resources may offer a more comprehensive understanding of the mechanisms. Second, due to the cross-sectional design, this study cannot infer causality from the results. It is plausible that the relationships among work–family conflict, hope, and burnout are reciprocal; for example, burnout may exacerbate perceptions of work–family conflict or further reduce hope. Future longitudinal or experimental research is needed to clarify the directionality and causal mechanisms of these relationships. These designs would provide stronger evidence for causal relationships. Third, this study might be influenced by social desirability bias due to the form of self-reported questionnaires, potentially leading participants to underreport or overreport the extent of studied variables, thereby influencing the observed associations. Although Harman’s single-factor test was conducted as a preliminary check for common method variance, this test has well-documented limitations and cannot fully rule out potential common method bias. Future studies could adopt more rigorous methodological approaches, such as temporal separation of measures, multi-source data, or longitudinal and diary-based designs, to minimize the potential bias associated with self-reported data. Additionally, as the present study focused on teachers from two large urban municipalities. Consequently, its findings may not be fully generalizable to teachers in other regions of China (e.g., central or rural areas). Future research could examine whether similar patterns emerge in other regions of China.

## 5. Conclusions

By integrating hope and a stress-is-enhancing mindset as complementary personal resources within COR theory, this study clarifies how cognitive appraisal and motivational resources interact under chronic work–family stress to influence teacher burnout. Hope was confirmed as a key mediator in the pathway from work–family conflict to burnout, while a stress-is-enhancing mindset buffered the depletion of hope and thereby mitigated the indirect impact on burnout. These findings refine the understanding of psychological capital and stress appraisal processes in the Chinese educational context. Practically, interventions that cultivate goal-directed hope and a stress-is-enhancing mindset may help reduce teacher burnout. Future research should examine other components of psychological capital and alternative moderation pathways to further elaborate the resource dynamics underlying burnout.

## Figures and Tables

**Table 1:** Demographic description.

Variables	Minimum	Maximum	Mean	SD	Number (%)
N					452 (100%)
Age	22	58	39.4	8.62	
Years of teaching	0	47	17.11	10.06	
Gender					
Female					355 (78.5%)
Male					97 (21.5%)
Educational level					
Senior high school					4 (0.9%)
Junior college					16 (3.5%)
Undergraduate					354 (78.3%)
Postgraduate					78 (17.3%)
Marital status					
Married					359 (79.4%)
Single					93 (20.6%)
Monthly income			9234.85	1807.16	
Health status	1	5	3.08	0.98	
Work–family conflict	1	5	3.31	0.66	
Hope	1	6	4.61	0.81	
Stress-is-enhancing mindset	1	5	3.04	0.52	
Burnout	1	7	3.14	1.01	

**Table 2:** Bivariate correlation analysis among variables.

	(1)	(2)	(3)	(4)	(5)	(6)	(7)	(8)	(9)
(1) Gender	-								
(2) Teaching years	−0.13 ***	-							
(3) Marital status	0.03	0.39 ***	-						
(4) Monthly income	−0.01 *	0.24 ***	0.11 *	-					
(5) Educational level	0.18 ***	−0.47 ***	−0.26 ***	−0.05	-				
(6) Health status	−0.06	−0.19 ***	−0.17 ***	−0.05	0.14 **	-			
(7) Work–family conflict	0.00	0.00	0.07	0.02	0.04	−0.32 ***	-		
(8) Stress-is-enhancing mindset	−0.04	0.02	0.06	0.05	0.00	0.21 ***	−0.24 ***	-	
(9) Hope	−0.04	0.12 *	0.10 *	0.16 **	−0.03	0.14 **	−0.15 **	0.31 ***	-
(10) Burnout	0.00	−0.09 *	−0.13 **	−0.11 *	0.07	−0.22 ***	0.49 ***	−0.36 ***	−0.44 ***

Note: * *p* < 0.05, ** *p* < 0.01, *** *p* < 0.001.

**Table 3:** Mediation Analysis of Hope in the Relationship Between Work–family Conflict and Burnout.

Variables	Model 1			Model 2		
	Outcome: Hope			Outcome: Burnout		
	B	Boot SE	Boot 95% CI	B	Boot SE	Boot 95% CI
Years of teaching	0.006	0.004	[−0.003, 0.014]	0.000	0.004	[−0.008, 0.009]
Married status	0.200 *	0.098	[0.004, 0.388]	−0.297 **	0.103	[−0.499, −0.093]
Monthly income	1.301	0.677	[−0.074, 2.518]	−0.563	0.424	[−1.396, 0.271]
Health status	0.125 **	0.049	[0.023, 0.215]	−0.067	0.043	[−0.152, 0.015]
Work–family Conflict	−0.142 *	0.076	[−0.297, −0.001]	0.657 ***	0.062	[0.530, 0.773]
Hope				−0.425 ***	0.052	[−0.533, −0.329]

Note: * *p* < 0.05, ** *p* < 0.01, *** *p* < 0.001. Direct effect = 0.657, SE = 0.061, 95%CI = [0.537, 0.776]. Indirect effect = 0.060, boot SE = 0.034, 95%boot CI = [0.001, 0.085].

**Table 4:** Moderated mediation Analysis.

Variables	Model 1			Model 2		
	Outcome: Hope			Outcome: Burnout		
	B	SE	95% CI	B	SE	95% CI
Years of teaching	0.005	0.004	[−0.003, 0.013]	−0.001	0.004	[−0.009, 0.008]
Married status	0.143	0.098	[−0.049, 0.335]	−0.271 **	0.100	[−0.467, −0.075]
Monthly income	1.210 **	0.406	[0.412, 2.008]	−0.496	0.418	[−1.317, 0.324]
Health status	0.078	0.041	[−0.002, 0.159]	0.056	0.042	[−0.138, 0.026]
Work–family conflict (WFC)	−0.598 *	0.255	[−1.100, −0.096]	0.152	0.262	[−0.363, 0.666]
Stress-is-enhancing mindset (SM)	−0.127	0.259	[−0.635, 0.381]	−0.736 **	0.263	[−1.254, −0.219]
Hope				−0.389 ***	0.049	[−0.486, −0.292]
WFC×SM	0.171 *	0.081	[0.011, 0.330]	0.152	0.083	[−0.012, 0.315]
Model summary						
F	10.752 ***			38.740 ***		
R^2^	0.150			0.421		

Note: * *p* < 0.05, ** *p* < 0.01, *** *p* < 0.001.

## Data Availability

The data presented in this study are available on request from the corresponding author due to ethical and institutional restrictions.

## Abbreviations

The following abbreviations are used in this manuscript:

WFC	Work–family conflict
COR	Conservation of Resources
FIW	Family interference with work
